# Implementing a New Diabetes Resource for Wisconsin Schools and Families

**Published:** 2005-10-15

**Authors:** Angela Nimsgern, Jenny Camponeschi

**Affiliations:** Wisconsin Department of Health and Family Services, Division of Public Health, Northern Region, Diabetes Prevention and Control Program; Wisconsin Department of Health and Family Services, Division of Public Health, Diabetes Prevention and Control Program, Madison, Wisc

## Abstract

**Background:**

Diabetes is one of the most common diseases in the nation. Students with diabetes face the daily task of balancing food, physical activity, and medication to survive. Teachers and school personnel often lack the knowledge needed to assist them.

**Context:**

An estimated 2647 schoolchildren in Wisconsin have diabetes. The Wisconsin Diabetes Prevention and Control Program frequently receives anecdotal reports from parents and diabetes educators on the care of children with diabetes in the schools; the program also manages requests for information on new diabetes-related equipment from school personnel.

**Methods:**

A statewide workgroup convened to develop *Children with Diabetes: A Resource Guide for Wisconsin Schools and Families, *aimed at improving the school staff's knowledge of diabetes and its management and their awareness of the benefits of maintaining glucose control. Training sessions for school professionals were developed and conducted around the state. All attendees were asked to complete an evaluation of the training. In addition, the workgroup included an evaluation form with each guide distributed and conducted a follow-up survey on the impact of the guide and changes to school policies.

**Consequences:**

Of the 762 people who attended training sessions, 631 (83%) completed the evaluation form. On questions about the training session's content, quality, organization, and appropriateness, responses averaged 4.42 points on a scale of 1 (poor) to 5 (excellent). More than 9713 resource guides were distributed to more than 1359 individuals; 58 recipients responded to the evaluation form included with the resource guide, with 57 (98%) of these indicating that they would recommend the guide to others. Preliminary results of the follow-up impact survey show that many positive changes have been implemented to improve the school environment for children with diabetes since the resource guide was implemented.

**Interpretation:**

This model of working with school professionals, health care practitioners, parents, and community organizations to create a resource guide with accompanying training sessions can be used in other states to accomplish similar goals of increasing knowledge about diabetes and improving social and policy environments.

## Background

Diabetes is one of the most common diseases in the nation. An estimated 210,000 Americans aged younger than 20 years, or approximately one in every 400 (0.26%), have diabetes (1). Type 2, the most common form of diabetes in the United States, is becoming more common among that population; however, the vast majority of Americans aged younger than 20 with diabetes have type 1 (1).

Diabetes causes blood glucose levels to be above normal. Type 1 diabetes is characterized by the absence of insulin and is managed through balancing food intake, daily physical activity, and insulin injections. Type 2 diabetes is characterized by the body's inability to produce enough insulin or use insulin properly. Type 2 diabetes is treated with diet, physical activity, and often oral medications, insulin injections, or both (2).

In 1993, the Diabetes Control and Complications Trial (DCCT) demonstrated that glycemic control in an intensively treated group of people with type 1 diabetes delayed the onset of microvascular complications (e.g., retinopathy, nephropathy, neuropathy) and slowed the progression of complications that already were present. The benefits of lower blood glucose levels were seen for all people regardless of age, sex, duration of diabetes, or history of poor diabetes control (3). Intensive diabetes management is most appropriate for people willing and able to monitor their blood glucose frequently throughout the day and adjust their insulin, food intake, and physical activity based on blood glucose test results. Any improvement in glycemic levels may help decrease the risk of complications.

The American Diabetes Association states that trained adults supporting children with diabetes are important to avoiding the hazards of hypoglycemia and to achieving the glycemic control required to decrease later risk for developing complications (4). In addition to the physiological benefits of maintaining glycemic control, it has been shown that lower hemoglobin A1c levels are linked with higher perceived quality of life among adolescents (5-7). Glycemic control is also associated with optimal academic performance (4).

According to Klingensmith et al, "To facilitate the appropriate care of the student with diabetes, school and day care personnel must have an understanding of diabetes and must be trained in its management and in the treatment of diabetes emergencies" (4). Teachers, day care staff, and other school personnel often lack the knowledge needed to assist children with diabetes.

A recent study of 105 children aged 6 to 14 years diagnosed with type 1 diabetes found that those children believed that teachers, nurses, and friends needed to improve their knowledge about diabetes (8). Children aged 11 to 14 years reported that their athletic coaches also needed more knowledge about diabetes. Increased knowledge may lead to optimal assistance and flexibility in allowing children to follow their medical regimens in the classroom. In addition, informed school personnel and friends would be alert to a child who appeared to be experiencing hypoglycemia. The study's researchers found that "children needed support in six key areas: educating staff, availability of supplies, teacher flexibility, help with hypoglycemic episodes, reminders to follow their regimens, and emotional support" (8).

## Context

There are 3189 public and private schools in Wisconsin with a total enrollment of 1,017,883 students (9). Based on the national prevalence rate of diabetes among people aged younger than 20 years (0.26%), we estimate that 2647 school children in Wisconsin have the disease (10). These students face the daily task of balancing food intake, physical activity, and medication — usually insulin — to survive with diabetes. When these three parts of their day are synchronized, the children are able to participate fully in all the activities of their peers. When one or more of the three are out of balance, children with diabetes can experience low or high levels of blood glucose. These abnormal levels can affect how they feel, behave, think, and learn and can even lead to hospitalization. In 2002, there were 769 diabetes-related hospitalizations among youths aged 5 to 18 years in Wisconsin, representing 2.56% of all hospitalizations for this age group (11).

The Wisconsin Diabetes Prevention and Control Program (WDPCP) frequently receives reports from parents, diabetes educators, and others on the care of children with diabetes in schools. Some anecdotal reports have included descriptions of children experiencing acute hypoglycemic events as they were being sent alone to the nurse's office for blood glucose testing when they were "feeling low," children being excluded from school events because of misunderstandings about the disease, and unnecessary delays in administering glucagon during extreme hypoglycemia. The WDPCP also frequently receives requests from school professionals for information on new equipment, such as insulin pens and pumps, and for information on new diabetes medications being used by their students.

## Methods

Responding to concerns about the care of children with diabetes in schools and recognizing the size and potential impact of the health care issues, three programs in Wisconsin —  the WDPCP, the Comprehensive School Health Program, and the Children with Special Health Care Needs Program — organized a workgroup to develop a resource for people working with children who have diabetes: *Children with Diabetes: A Resource Guide for Wisconsin Schools and Families*.

Workgroup members represented key statewide organizations and individuals committed to improving diabetes care for children in schools throughout Wisconsin (Table 1). They reviewed existing documents written with goals similar to their own; the *Resource Guide for Families of Children with Diabetes* and the *Diabetes Resource Guide for Schools*, both produced by the New York Diabetes Prevention and Control Program, served as the primary models for the Wisconsin resource guide.

**Figure 1:**
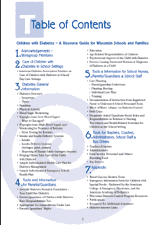
View the *Children with Diabetes: A Resource Guide for Wisconsin Schools and Families* Table of Contents (PDF 107K)

The initial group of 48 partners organized into subgroups to draft individual sections of the resource guide. The final guide includes information on disease management, legal issues, diabetes management and monitoring forms, and tools such as field trip tips and suggested agendas for planning meetings. It also describes the roles of parents, school nurses, teachers, coaches, administrators, food-service staff, and bus drivers (Sidebar).

Of special significance to the workgroup was the acknowledgment that Wisconsin public school districts are not required to employ a school nurse. The lack of school nurses underscores the importance of having academic and administrative staff members who understand diabetes and how best to assist children with blood glucose monitoring and medication administration, including managing glucose levels that are outside of target ranges (2). In a "Tools and Information" section of the resource guide, the workgroup addresses 28 frequently asked questions about roles and responsibilities related to nursing procedures and health-related activities.

An additional subgroup was formed to plan promotion, distribution, and training for the resource guide. To assist with promotion and training, the WDPCP contracted with a certified diabetes educator who had strong experience working with adolescents and their schools. The WDPCP staff and the diabetes educator developed a training program for school professionals. Suggestions for training content and target audience groups were solicited from workgroup members. Between June 2002 and April 2003, participants traveled to a central location, such as the regional Cooperative Education Service Association (CESA) building or a conference center, for in-person presentations conducted by the diabetes educator that included hands-on exercises and equipment samples. The training sessions provided an overview of diabetes in school-aged children and addressed disease management, nutritional considerations, laws, and policy issues. They were conducted during the school day and ranged from half-day sessions for some large school districts and CESA districts to hour-long breakout sessions at statewide conferences of school professionals. The diabetes educator conducted 21 total sessions for 762 school and support staff (Table 2).

Workgroup members also obtained endorsement of the resource guide from their own organizations and promoted it through their organizations' newsletters, Web sites, and electronic mailing lists. The WDPCP provided sample newsletter articles and Web site links to workgroup members.

The WDPCP evaluated *Children with Diabetes: A Resource Guide for Wisconsin Schools and Families* in the following four ways: 1) by asking all training session attendees to complete an evaluation; 2) by tracking promotional efforts such as exhibit booths and news postings through verbal, written, and e-mail reports from workgroup members and other WDPCP partner organizations; 3) by including an evaluation form with every resource guide distributed; and 4) by conducting a follow-up survey among resource guide recipients on the impact of the resource guide on school policies.

## Consequences

### Training sessions

Of the 762 people who attended training sessions, 631 (83%) completed the evaluation form (Appendix A). The majority of evaluation participants were school nurses (n = 243); the second largest group was food-service workers (n = 150). Of the 631 evaluation participants, 459 (73%) stated that they had worked with a child with diabetes in the school setting; 143 (23%) had not worked with a child with diabetes; 15 (2%) did not know if they had; and 14 (2%) did not respond to the question.

On questions about the training session's content, quality, organization, and appropriateness, responses averaged 4.42 points on a scale of 1 (poor) to 5 (excellent). Four questions targeted the value of the training; respondents rated the sessions an average of 3.76 points on a scale of 1 (strongly disagree) to 4 (strongly agree) for these questions.

Open-ended questions allowed respondents to share information about the aspect of the training that was most helpful and least helpful, and about any change they would suggest to improve the training. Revisions to the training format (e.g., increased length, increased networking opportunities, more information on certain subjects) were made to subsequent sessions based on these responses. Responses were not tracked by length of training attended, so no comparisons can be made between the impact of the 1-hour sessions and the longer sessions.

### Promotional efforts

Workgroup members have reported activities such as giving presentations to their organization, distributing order forms or resource guides to colleagues, providing exhibits or announcements at conferences, offering Web site links, publishing newsletter articles, and mailing promotional materials. We counted five direct mailings, eight Web site postings, 14 electronic mailing list or newsletter articles, and 33 face-to-face presentations.

Evaluation information on promotional activities provided by workgroup members was sparse: only 25 reports of promotional activities were received. We expected the difference in response rates between the onsite training evaluations and the mailed or faxed reporting of promotional activities through *Report of Action* forms (Appendix B). The low response rate, however, limits the ability to draw conclusions about the effectiveness of the promotional efforts. For future efforts, we will consider sending a subsequent mailing requesting feedback in exchange for an incentive gift to raise response rates.

### Resource guide evaluation form

More than 9713 resource guides were distributed. A sample of guides included an evaluation form (Appendix C). Only 58 recipients of the resource guide returned evaluation forms; 57 of these respondents indicated they would recommend the guide to others.

### Follow-up impact survey

A follow-up survey was sent to 1359 individuals for whom we had mailing addresses and who had received the resource guide. This impact survey sought to determine the number of schools that have made changes to improve the school environment for children with diabetes and what those changes are (Appendix D). Preliminary results indicate that of the 331 respondents, 34 (10%) reported that they frequently referred to the resource guide, 73 (22%) referred to it several times, 158 (48%) referred to it a few times, and 63 (19%) never referred to it. Of the survey respondents, 13 (4%) reported that they frequently referred parents, colleagues, or both to the resource guide, 59 (18%) referred others several times, 143 (43%) referred others a few times, and 109 (33%) never referred others.

The survey's open-ended questions demonstrate that policy and procedure changes have occurred in schools that received the resource guide. Following are examples of the changes noted by respondents: incorporating healthy snacks into the school day, eliminating soda machines and the sale of candy during the school day, modifying field trip procedures, providing information on the carbohydrate content of cafeteria items, and allowing blood glucose tests in the classroom. The impact survey also indicated that schools have made changes to improve the school environment for children with other chronic conditions, such as asthma and seizure disorders.

We plan to provide updates to the resource guide. We will update information on the types of insulin and insulin-delivery systems that youths are using because this information becomes outdated quickly. We produced the resource guide in a loose-leaf notebook using 3-hole–punched paper to allow for periodic updates and addition of local information. New information will be based on feedback from resource guide recipients and on new developments in diabetes-care practices, equipment, and school policy. We anticipate an expanded discussion of the incidence and management of type 2 diabetes in youths.

## Interpretation

The development and implementation of the *Children with Diabetes: A Resource Guide for Wisconsin Schools and Families* was a valuable process that brought a needed reference to schools throughout Wisconsin and assisted schools in making changes to improve the school environment for children with diabetes. This model of establishing new partnerships to create a useful document with accompanying training sessions can be replicated in other states to accomplish similar goals. Bringing individuals and organizations into the development process increased the support for the resource guide and greatly enhanced the reach of our implementation efforts. Although there was a strong advantage to incorporating Wisconsin-specific information into the resource guide, it is not necessary for every state to develop its own guide. Since we developed the Wisconsin resource guide, the National Diabetes Education Program (NDEP) released *Helping the Student with Diabetes Succeed: A Guide for School Personnel*. This document, published in 2003, includes a diabetes primer and glossary; action steps for key school personnel, parents, and students; sample medical management and emergency action plans; review of school responsibilities under federal laws; and copier-ready handouts (12). Localities can review the national resource as well as consult with their state's department of education for information on how state laws may affect diabetes care.

When the need for a Wisconsin resource guide became apparent, the NDEP guide was not yet available, thus leading partners to create the Wisconsin guide. Furthermore, the WDPCP successfully developed and implemented the *Wisconsin Essential Diabetes Mellitus Care Guidelines* and believed that the creation and implementation of the resource guide would have similar success. It was also important to our partners to create a guide that was applicable to the residents and laws of Wisconsin.

Although the resource guide cannot directly change the day-to-day practice within every school, it can be used as a resource for school personnel. The resource guide helps to identify concrete solutions to deliver the best diabetes management care in schools.

We continue to work with school professionals and parents to improve the school environment for children with or at risk for diabetes. The WDPCP staff regularly provides updates on diabetes-related research findings, medications, and equipment. We also work with statewide and local groups targeting physical activity and nutrition in the schools to enhance diabetes management for children already diagnosed and to improve the environment for those at risk of developing type 2 diabetes. These efforts, and those of collaborating organizations supported by the resource guide, can lead to improved understanding between youths with diabetes and the adults in positions to help them. This improved partnership will enhance the school environment for these youths and potentially lead to improved glycemic control and reduced long-term complications.

## Figures and Tables

**Table 1:** Workgroup Partners, *Children with Diabetes: A Resource Guide for Wisconsin Schools and Families*

**Organizations represented in workgroup**
American Diabetes Association of Wisconsin
Association of Wisconsin School Administrators
American Academy of Pediatrics — Wisconsin Chapter
Brown County Children with Disabilities Education Board
Cooperative Education Service Association
Children's Hospital of Wisconsin
Gundersen Lutheran Pediatric Clinic
Juvenile Diabetes Research Foundation
Legislative Liaison
Local public health departments (2 groups)
Local school districts (7 groups)
Marshfield Clinic Pediatric Endocrinology Clinic
Parents (2 people)
Pharmacy Society of Wisconsin
School Para-Professionals Organization
Students
University of Wisconsin Children's Hospital
Wisconsin Association of School Boards
Wisconsin Association of School District Administrators
Wisconsin Association of School Nurses
Wisconsin Board of Nursing
Wisconsin Child Care Improvement Project
Wisconsin Dietetic Association
Wisconsin Education Association Council
Wisconsin Nurses Association
Wisconsin Parent–Teacher Association
Wisconsin School Bus Association
Wisconsin Department of Health and Family Services, Division of Public Health: Bureau of Community Health Promotion Children with Special Health Care Needs Program Comprehensive School Health Program Diabetes Prevention and Control Program Emerging Medical Sciences for Children Program
Wisconsin Department of Public Instruction, School Nursing/Health Programs

**Table 2:** Implementation and Promotion Events for *Children with Diabetes: A Resource Guide for Wisconsin Schools and Families*

**Groups receiving diabetes training through sessions at larger conferences or at specially scheduled events**
Cooperative Education Service associations (10 of Wisconsin's 12)
Milwaukee Health Department
Milwaukee Public School Food Service
Public health nurses/school nurses (3 Wisconsin groups)
Wisconsin Association of School Nurses^a^,^b^ (3 regional groups and at state convention)
Wisconsin School Bus Association^a^
Wisconsin School Food Services Association^a^

**Groups receiving special promotion through exhibits or brief presentations (typically at annual statewide meetings)**

American Diabetes Association of Wisconsin^b^
Association of Wisconsin School Administrators
Children with Special Health Care Needs, Southern Regional Center
Juvenile Diabetes Research Foundation^b^
Local parents group in central Wisconsin
Regional groups of diabetes educators
Staffs of local schools and health care agencies (9 groups)
UW-Milwaukee School of Nursing/Southeastern Public Health Nurses
Wisconsin Association of School Business Officials
Wisconsin Academy of Physician's Assistants
Wisconsin Association of School Boards
Wisconsin Association of School District Administrators
Wisconsin Council of Religious and Independent Schools
Wisconsin Diabetes Advisory Group
Wisconsin Dietetic Association^b^
Wisconsin Early Childhood Association
Wisconsin Education Association Council
Wisconsin Parent–Teacher Association^b^
Wisconsin Public Health Association
Wisconsin public health nurses

**Organizations promoting the resource guide through own Web sites, electronic mailing lists, or newsletters**

Wisconsin Child Care Improvement Project
Wisconsin teachers
Wisconsin Title V (Maternal and Child Health) Block Grant Providers
Wisconsin Medical Society
Children's Hospital of Wisconsin community
Diabetes education programs recognized by American Diabetes Association
Wisconsin Department of Public Instruction
Children with Special Health Care Needs, regional centers
Wisconsin Education Association Trust
Children's Health Alliance network

a Group received both a diabetes training event and special promotion.

b Group received both a news posting and special promotion or training.

## Appendices

### APPENDIX A: CHILDREN WITH DIABETES TRAINING EVALUATION FORM

***Children with Diabetes: A Resource Guide for Wisconsin Schools and Families***

**Training Session Evaluation**

Thank you for attending the "Caring for a Child with Diabetes in Your School" training.

Please help us improve future training sessions by sharing your comments about this session.

We appreciate your time and consideration.

**Please identify your role in working with children with diabetes in the school setting:**

*(Check all that apply)*

□ Bus Driver	□ School Administrator
□ Food Service Worker	□ School Nurse
□ Parent/Guardian	□ Teacher
□ Physical Education Teacher	□ Other (please specify) _____________________________

**Have you worked with a child that has diabetes in the school setting?**

□ Yes
□ No
□ Don't know/not sure

**Table:**

	**Excellent**	**Above Average**	**Average**	**Below Average**	**Poor**
(1) Content/subject matter	5	4	3	2	1
(2) Overall quality of training	5	4	3	2	1
(3) Overall organization of training	5	4	3	2	1
(4) Appropriateness of training strategies *(presentations, demonstrations, handouts, question and answer session, interaction, etc.)*	5	4	3	2	1
(5) Application to my role in working with children with diabetes	5	4	3	2	1
(6) Content of main speaker's presentation	5	4	3	2	1
(7) Handout materials	5	4	3	2	1
(8) Question and answer period	5	4	3	2	1
(9) Length of overall training	5	4	3	2	1
(10) Facility meeting room	5	4	3	2	1

**Table:**

	**Strongly Agree**	**Partially Agree**	**Partially Disagree**	**Strongly Disagree**
(11) This training covered what I expected it would cover.	4	3	2	1
(12) I feel more knowledgeable about children with diabetes in the school setting.	4	3	2	1
(13) I understand my role in assisting children with diabetes in the school setting.	4	3	2	1
(14) I would recommend this training to another person with a role similar to mine.	4	3	2	1

(15) What aspect of this training was most helpful?  ________________________________________

(16) What aspect of this training was least helpful?  ________________________________________

(17) What change(s) would you make to this training?  ________________________________________

## APPENDIX B: REPORT OF ACTION*CHILDREN WITH DIABETES: A RESOURCE GUIDE FOR WISCONSIN SCHOOLS AND FAMILIES*

The Wisconsin Diabetes Prevention and Control Program (WI DPCP) recently completed and distributed *Children with Diabetes: A Resource Guide for Wisconsin Schools and Families*. To assist in evaluation of the *Resource Guide*, we are interested in activities you and your organization have done to promote the *Resource Guide*.

Please complete this brief form and return to the WI DPCP by **Friday, June 6, 2003**. A self-addressed stamped envelope is enclosed for your convenience or you may fax it to (608) 266-8925.

**What activities have you or your organization done to promote the *Resource Guide*? *(Check all that apply)***

- □ Given presentation(s) to my organization
- □ Distributed *Resource Guide* order forms to my organization
- □ Completed a mailing to organization members
- □ Included an article in organization's newsletter *(please attach a copy, if available)*
- □ Issued a press release *(please attach a copy, if available)*
- □ Added *Resource Guide* link to organization's website
- □ Shared at conferences my organization attends
- □ Held or sponsored training session(s)
- □ Other *(Please specify)* ________________________________________

**Please provide any additional comments or suggestions about the *Resource Guide* distribution, implementation and/or trainings. You may also include feedback regarding the content of the *Resource Guide*.**

________________________________________

As a condition of federal funding, the WI DPCP must obtain "in-kind" contributions. The DPCP partners' activities are included in this contribution. If your organization has contributed through activities to promote *Children with Diabetes: A Resource Guide for Wisconsin Schools and Families*, please record that information below.

Name:  ________________________________________

Organization: ________________________________________

**Table:**

**Date**	**Description of Activity**	**In-kind Contribution *(Estimate)***
		

***Thank you for taking the time to complete this Report of Action. Please return this form to the Wisconsin Diabetes Prevention and Control Program in the enclosed envelope or fax to (608) 266-8925. If you have questions, please contact Judy Wing at (608) 261-6855. Thank you!***

## APPENDIX C: CHILDREN WITH DIABETES EVALUATION OF RESOURCE GUIDE

***Children with Diabetes: A Resource Guide for Wisconsin Schools and Families***

**Evaluation of Resource Guide**

*Please help us improve future versions of the resource guide by sharing your comments.*

**Please identify your role in working with children with diabetes in the school setting:** *(Check all that apply)*

□ Bus Driver	□ School Administrator
□ Food Service Worker	□ School Nurse
□ Parent/Guardian	□ Teacher
□ Physical Education Teacher	□ Other (*please specify*) ______________________________

**Have you worked with a child with diabetes in the school setting?**

□ Yes
□ No
□ Don't know/not sure

**Table:**

** **	**Strongly Agree**	**Partially Agree**	**Partially Disagree**	**Strongly Disagree**	**Not Applicable**
I was able to find the information that I needed in the resource guide	5	4	3	2	1
The information I was looking for in the resource guide was easy to find	5	4	3	2	1
I learned something new from the resource guide	5	4	3	2	1
The resource guide is a good source for future questions or concerns I might have about a child with diabetes	5	4	3	2	1
The resource guide is a good source of information even if I do not have a specific question about a child with diabetes	5	4	3	2	1
I expect to use the resource guide again in the future	5	4	3	2	1
If a colleague mentioned he/she knew a child with diabetes, I would refer him/her to this guide as a resource	5	4	3	2	1

(8) What part(s) of this resource guide was most helpful?  ________________________________________

(9) What part(s) of this resource guide was least helpful?  ________________________________________

(10) What change(s) would you make to this resource guide?  ________________________________________

(11) Thinking of your needs or interests, what topics would you recommend for future editions of this resource guide and/or Diabetes in the School Setting professional or public awareness activities?  ________________________________________

(12) Would you recommend this resource guide to others?

□ Yes  □ No □ Don't know/not sure

***Thank you for taking the time to complete this evaluation. Please fax this completed evaluation to (608) 266-8925 or mail to Judy Wing, Diabetes Control Program, 1 W Wilson, PO Box 2659, Madison, WI 53701-2659.***

## APPENDIX D: CHILDREN WITH DIABETES IMPACT SURVEY

***Children with Diabetes: A Resource Guide for Wisconsin Schools and Families***

In the past, you received a copy of *Children with Diabetes: A Resource Guide for Wisconsin Schools and Families*. The guide will be updated in 2005. The Wisconsin Diabetes Prevention and Control Program would like to learn how this guide is being used, what policy changes (if any) have been made, and suggestions on how to improve or expand the guide. Please take a few moments to complete this brief survey and return it by **July 1, 2005**.

In appreciation for completing the survey you will be sent a pedometer and a copy of the updated Essential Diabetes Mellitus Care Guidelines. Also, we will notify you by e-mail when the revised resource guide is available.

**1. What type of organization do you represent?**

- □ School (Circle type)   *Elementary      Middle      High School*
- □ Local Health Department
- □ Cooperative Educational Service Agency
- □ Other (Please specify)  ________________________________________

**2. What is your role within this organization?** ________________________________________

**3. Do you still have your copy of the *Children with Diabetes* *Resource Guide*?**

- □ Yes
- □ No
- □ Don't Know

**4. How often have you referred to the *Children with Diabetes* *Resource Guide*?**

- □ Never
- □ A few times
- □ Several times
- □ Frequently

**5. How often have you referred colleagues/parents to the *Children with Diabetes* *Resource Guide*?**

- □ Never
- □ A few times
- □ Several times
- □ Frequently

**6. Did you attend a Diabetes Prevention and Control Program or Cooperative Educational Service Agency-sponsored training regarding the** ***Children with Diabetes Resource Guide*?**

- □ Yes
- □ No
- □ Don't remember

**7. Did you attend any educational training sessions on diabetes in the schools at a statewide professional conference in the past year?**

- □ Yes
- □ No
- □ Don't remember

**8. How has the *Children with Diabetes* *Resource Guide* been used in your organization? **________________________________________

**9. Please identify all areas where policy or procedure changes have been made to improve the school environment for children with diabetes. **

- □ School Nurse/Health Services
- □ Physical Education
- □ Food Service
- □ Emergency Procedures (fire, weather, bomb, security threats, etc.)
- □ Extracurricular Activities
- □ School Sports
- □ School Sponsored Events
- □ Recess/Nonacademic Time
- □ Personal Health Emergencies
- □ Other (Please specify) ________________________________________
- **Provide a brief summary of the changes made in each area you identified.**
  ________________________________________

**10. What is total estimated enrollment of school(s) implementing these changes?**

- □ <100
- □ 100-499
- □ 500-999
- □ 1000-1500
- □ >1500

**11. What changes (if any) have been made that improve the school environment for children with *other* health conditions (e.g., asthma, seizure disorders) as a result of information in the** ***Children with Diabetes Resource Guide*? **________________________________________

**12. Please rate the usefulness of each section of the *Children with Diabetes Resource* *Guide* by indicating your response below.**

**Table:**

	**Extremely Useful**	**Somewhat Useful**	**Not Useful**	**No Opinion/Not Used**
American Diabetes Association Position Statement	3	2	1	N/A
Diabetes General Information	3	2	1	N/A
Tools and Information for Parents/Guardians	3	2	1	N/A
Tools and Information for School Nurses, Parents/Guardians and School Staff	3	2	1	N/A
Tools and Information for Teachers, Coaches, Administrators, School Staff, and Bus Drivers	3	2	1	N/A
Appendices	3	2	1	N/A

**13. What technical assistance is needed for schools to further implement changes based on the information in the *Children with Diabetes Resource Guide*?**

________________________________________

**14. What additional training about diabetes (if any) would be beneficial to your school or to you personally?**

________________________________________

**15. The guide will be updated in 2005. Please suggest any changes or additions that would make it more helpful/useful.**

________________________________________

***Thank you for taking the time to complete this survey! Fax this form (please remember all pages) to Judy Wing at the Diabetes Prevention and Control Program at (608) 266-8925.***

Please fill out your contact information below. This information will remain confidential and will only be used to send the aforementioned materials.

Name ______________________________________

Address ____________________________________

City/State/Zip _______________________________

E-mail Address _____________________________

For more information on the Wisconsin Diabetes Prevention and Control Program, please visit our Web site at: http://dhfs.wisconsin.gov/health/diabetes/ or call (608) 261-6855.
