# Supplementary material for: Implementing a New Diabetes Resource for Wisconsin Schools and Families
Source: Prev Chronic Dis. 2005 Oct 15;2(Spec No):A11. (PMC1459474)
Supplement: Supplementary file 1 [file 05_0081_01.pdf]

Table of Contents

Children with Diabetes – A Resource Guide for Wisconsin Schools and Families

1. Acknowledgements – Workgroup Members

2. Care of Children with Diabetes in School Settings

- American Diabetes Association Position on Care of Children with Diabetes in School/ Day Care Settings

3. Diabetes General Information

- Diabetes Overview
  - Symptoms
  - Types
- Nutrition
- Physical Activity
- Blood Sugar Monitoring
- Hypoglycemia (Low Blood Sugar)
  - What is Glucagon?
- Hyperglycemia (High Blood Sugar) and Monitoring for Presence of Ketones
  - Urine Testing for Ketones
- Insulin and Insulin Delivery Systems
  - Insulin
  - Insulin Delivery Systems (syringes, pens, pumps)
  - Disposing of Sharps Safely (syringes, lancets)
- Helping Others Take Care of the Child with Diabetes
- Sample Individualized Health Care Plan for Diabetes Management
- Sample Individualized Emergency School Health Plan

4. Tools and Information for Parents/Guardians

- Juvenile Diabetes Research Foundation – Your Child Has Diabetes
- Parents/guardians of Children with Diabetes Have Responsibilities Too
- Appropriate Accommodations Under Law
- Parents'/guardians' Rights
- Education
- Age-Related Responsibilities of Children
- Psychosocial Aspects of the Child with Diabetes
- Factors Causing Emotional Distress at Diagnosis of Diabetes in a Child

5. Tools & Information for School Nurses, Parents/Guardians & School Staff

- Care Planning
  - Parent/guardian Conference
  - Planning Meeting
  - Individual Care Plan
  - Training
- Documentation of Instruction from Registered Nurse to Unlicensed School Personnel Form
- Effect of Illnes s/Injury on Diabetes Control
- Field Trips
- Frequently Asked Questions About Roles and Responsibilities in Relation to Nursing Procedures and Health-Related Activities for Children in the School Setting

6. Tools for Teachers, Coaches, Administrators, School Staff & Bus Drivers

- Teachers/Coaches
- Administrators
- Food Service Personnel and Others Providing Food
- Bus Drivers

7. Appendix

- Blood Glucose Monitor Form
- Emergency Information Form for Children with Special Needs – Endorsed by the American College of Emergency Physicians, and the American Academy of Pediatrics
- Wisconsin Diabetes Control Program Resources
- Publications
- Resources for Additional Assistance
- Diabetes Internet Links
